# The Engineering Side of Hospital Work

**Published:** 1905-05-13

**Authors:** 


### HOSPITAL ADMINISTRATION.

### CONSTRUCTION AND ECONOMICS.

### THE ENGINEERING SIDE OF HOSPITAL WORK.

(*Continued from page* 110.)

XII.—ELECTRICAL APPARATUS.

Electricity is employed very largely in hospitals, and it is more than probable that as time goes on its use will steadily increase. As the large hospitals are virtually small towns, with a good deal more life than the majority of these have, it is only natural that the great convenience of electricity should commend itself to those on whom the responsibility of administration rests, and in addition, especially since the discovery of the Roentgen rays, the value of electricity is being more and more recognised in both medical and surgical work. Electricity is used for lighting, for telephonic communication, for the distribution of power, as well as in medicine and surgery. The writer believes that the possibilities of electricity in medicine have hardly been realised by the medical profession, for the reason, amongst others, that its properties have not been understood, and he proposes, therefore, to give a *résumé* of those properties, and to lead from them to the apparatus in use, or that may be used for medical and surgical purposes. The study of electricity, the writer believes, has been somewhat hampered, on the part of members of the medical profession, by the fact that there are apparently two electricities—that taught in the schools and colleges, and that used by practical engineers. Forty years ago, when the late Professor Fleeming Jenkin wrote what was then the standard book on Electricity and Magnetism, he made the same complaint, coupled with the further one that the electricity of the practical engineer was then more scientific than that taught by the schools; and this complaint also might be made to-day. The electricity as known to the majority of professors and school teachers is often very inaccurate, from the theoretical point of view, and the reason is, those who teach it have rarely had the advantage, one which members of the medical profession will appreciate, of bringing the theories they teach to the test of practice. And hence it has followed that very grave mistakes have been made, often leading to the waste of large sums of money, by the propagation of ideas, founded on crude laboratory experiments, by men whose names as teachers of electricity are household words throughout a large portion of the civilised world. Members of the medical profession will appreciate, perhaps more than those of any other profession, the enormous importance of a knowledge of all the factors in the problem to be solved, and they, of all others, will realise the very great difficulty there is in obtaining all the factors. To the modern engineer, as to the modern physician and surgeon, there is only one safe guide, and that is a thorough knowledge of the principles of the science upon which his work depends, checked by constant appeal to practical work. It is on these lines the present writer has prepared these articles, and it is on these lines, more particularly, that he proposes to deal with electrical apparatus. He wishes it to be understood that he writes as an engineer who has a knowledge of the anatomy of the human body, and of the laws of physiology, but he merely proposes to state the laws and their effects as they appear to an engineer, leaving the application of the information he places before them to the members of the medical profession.

In the first place the writer would point out that, however generated, there is only one electricity, just as there is only one heat, though the form in which the electricity is manifest may, and does, vary very widely indeed, just as the form in which heat is manifest varies. Further, with the form in which electricity is manifest its properties vary. That is to say, while all the properties of electricity are common to all forms in which it may appear, some properties become masked by the conditions present with certain forms, while other properties are magnified very much. Large figures in any physical force always, apparently, bring their own special properties, while really the special properties are merely the result of the high values of the particular factors. High temperatures bring into prominence certain properties of metals and other substances merely because of the high temperatures. Similarly high pressures with electrical apparatus bring into prominence certain special features, such as the ability to throw sparks across intervals of air and through other substances, and the mechanical properties that will be referred to. The fact that a current is alternating instead of always passing in one direction again brings into prominence another very important group of properties in connection with magnetism and induction that are apparently absent with continuous currents, though really only weakened, and these properties develop further peculiarities when the alternations are at a very high rate—say a million per second—as in the well-known high-frequency treatment.

One reason for the fact that electricity has been taught so differently by the schools and by practical engineers is, it has been studied from points of view so different as to give the appearance of two different forces. In the college laboratory the favourite study has been electricity as it is generated by frictional and similar apparatus, in which the pressures created have always been very high, but the quantity of electricity generated very small indeed. On the other hand, the practical engineer has dealt with currents of comparatively large quantity, but very low pressure. Naturally, therefore, the two sets of observers have recorded views as of different forces, where really they should have recorded different views of the same phenomena. As, however, the practical side has developed, and as the school side has made discoveries of great importance, the two branches have drawn nearer and nearer together, so that the leading minds in both view the whole phenomena as they should be viewed—from all sides, not from any particular standpoint. On the side of the practical engineers, larger and larger quanties of power to be distributed over greater and greater distances have obliged them to use higher and higher pressures till at the present time in Mexico they are using a pressure 70,000 volts, that is quite within the region hitherto dominated by the college laboratory, while pressures of tens of thousands of volts are used in other parts of the world. And hence the practical engineer has met with many of the phenomena hitherto seen only in the laboratory in his practical work, but on a grand scale. On the very high-pressure distribution lines in America, for instance, where the pressures named above and others approaching them are used, the beautiful glow discharge of the frictional electrical machine is seen at night emanating from the transmission wires, and is not only seen, but takes toll of the power transmitted. The study of electricity from the purely high-pressure point of view has led to the very important discoveries in connection with the behaviour of the discharge *in vacuo*, and in air under the high pressures. It has also led to the discovery of the fact that the discharge of a lightning flash is not a simple passage of a single current, but a succession of currents in violent oscillation; perhaps one of the most important discoveries of modern times.

But the two points of view from which it has been studied have led to the enunciation of two different theories of electricity. In the early days of electricity we had first the two-fluid theory based upon the fact that apparently different properties were given to different substances by rubbing them with substances of one group, while opposite properties were imparted by rubbing the same substances with another group, a striking instance of the masking of certain properties generally by the prominence of certain other properties. The two-fluid theory, under which the peculiar properties noted were ascribed to the generation or separation of two imponderable fluids, gave way, under the stress of the fact that the opposite properties could be obtained from the same substances at will, to a single fluid theory, by which the earth was supposed to be an infinite reservoir of a single imponderable fluid, that might be rendered apparent by certain processes, such as galvanic action, friction, and others, and that could always be delivered to and obtained from the earth in infinite quantities. But it was discovered by practical engineers, that the earth is a conductor and behaves exactly like any other conductor, and that when currents were supposed to be delivered to earth, and swallowed up in the infinite reservoir, they had merely taken a path, often very tortuous and very long, through portions of the earth's crust, back to the point from which they set out. Currents which were sent from London to work telegraph instruments at Manchester or Birmingham, for instance, were found returning by way of Glasgow. Hence the single fluid theory fell into abeyance, and its place has been taken, among practical engineers, mainly by a theory which is the natural corollary of the mechanical or molecular theory of heat. It was a long time before this theory of heat, which was again a natural consequence of the upsetting of the corpuscular theory of light, was accepted. In Newton's time, it will be remembered, light was supposed to consist of material bodies projected towards us at a very high velocity from the sun. Young's wave theory, in which light was supposed to come to us in waves, similar to those we see when we throw a stone into the water, upset the corpuscular theory, and is the accepted theory at the present time. But the wave theory necessitated a medium in which the waves should be propagated, and hence the invention, or discovery, of the ether. But as the connection between heat and light was seen to be very intimate, it followed that heat was also a wave-motion in the ether, and the lengths of both heat and light waves have since been measured. But the connection between electricity and heat is at least as close as between heat and light. In both cases the expenditure of a certain quantity of energy in a certain way leads first to the creation of one of the physical forces, then, as the rate at which the energy is expended is increased, to a second, and then to a third. Thus, if a copper or other conductor have a current of electricity passed through it, at first a small quantity of heat is generated, giving off heat-waves from the conductor as from any heated body that has not reached the temperature at which red rays are emitted. As the strength of the current is increased—in other words, as the rate of expenditure of energy is increased—the copper becomes first red, then yellow, then white, and is then fused, sometimes again changing its colour in fusion, light being given out when the visible rays are reached. Hence it appeared to the thinking minds among practical engineers and to some of the professors that electricity was also a wave motion in the ether, just as heat and light are accepted to be. A natural extension of the theory carried the ether into all substances. A fluid that fills all space could also occupy interstices, such as we know to exist in apparently solid bodies, as metals and other substances, and the logical sequence was, that the electrical actions, with their extensions, the magnetic actions, took place in the ether which pervades all space and all substances. The discovery by Heinrich Hertz in 1888, upon which wireless telegraphy has been built, of the fact that electric waves did actually pass in the ether, and were capable of being measured, reflected, and refracted, very strongly confirmed the view that had been gradually forming in the minds of those who were studying the subject, and the argument was apparently clinched by some very delicate experiments carried out by Sir Arthur Rücker, and exhibited at the meeting of the British Association at Cardiff in 1891. One of the properties of heat is known to be the expansion and contraction of bodies, in definite proportion to the increase or decrease of temperature. Sir Arthur Rücker showed, by a very beautiful and very delicate experiment, that a glass plate subject to an influence similar to that present when heat expands a body, but due to electricity, would be compressed, the compression of the plate being due to the expansion of the conductors fixed on its edge, to which an electrical pressure was imparted. On the other hand, the study of the behaviour of electrically-charged bodies *in vacuo* has led to a very different theory, starting from the discoveries preceding Roentgen's, which the writer will give in the next article. He has entered fully into the subject, because he believes it will render it easier for his readers to follow the working of the laws, and where they lead, in the action of electricity in and upon the human body.

